# Impact of ranolazine on myocardial metabolic ischemia detected by phosphorus-31 magnetic resonance spectroscopy

**DOI:** 10.1186/1532-429X-18-S1-P97

**Published:** 2016-01-27

**Authors:** Gerald M Pohost, Heewon Kim, Gabriel Vorobiof, Laurn Contreras, Hooman Madyoon, Jeffrey Helfenstein, Norman Lepor

**Affiliations:** 1 USC, Los Angeles, CA USA; 2 Imaging, Westside Medical, Los Angeles, CA USA; 3 Medicine, Geffen School of Medicine at UCLA, Los Angeles, CA USA; 4 Westside Medical Associates of Los Angeles, Los Angeles, CA USA

## Background

Ranolazine (RAN) is a novel late sodium current inhibitor, effective in treating angina pectoris in patients with chronic stable CAD. Its therapeutic effectiveness is not well understood. It is thought to reduce myocardial energy utilization by enhancing diastolic relaxation and by increasing myocardial blood flow. The purpose of the present study is to define the mechanism for the effectiveness of RAN. We applied myocardial phosphorus-31 magnetic resonance spectroscopy (P-31 MRS) at rest and during handgrip stress to demonstrate the relationship between RAN treatment and stress-induced energy utilization with myocardial ischemia.

## Methods

Patients with angina pectoris defined by the presence of sub sternal pain, associated with stress, and effectively reduced with rest (or nitroglycerine), and with evidence of "ischemia" by conventional imaging approaches including: stress SPECT, PET or echocardiographic studies; or the presence of >70% diameter stenosis on conventional or CT coronary angiography were enrolled. RAN was administered in doses of 500 mg twice daily for 2 weeks added to existing medical therapy, and titrated to 1000 mg twice daily for 2 weeks. All subjects were evaluated at the start and upon completion of the study for the presence and severity of angina pectoris using the Seattle Angina Questionnaire (SAQ).

Prior to and after maximal RAN dose titration, a P-31 MRS study was performed at rest and during isometric hand-grip stress of moderate intensity. With P-31 MRS, myocardial metabolic ischemia was defined as an abnormal decrease of phosphocreatine:ATP ratio (PCr/ATP) during handgrip stress.

## Results

Nine of 16 subjects were identified as having metabolic ischemia by P-31 MRS prior to RAN. At 4 week follow-up, five of the nine P-31 studies (56%) with metabolic ischemia at baseline demonstrated evidence of improvement in the myocardial HEP response (p = 0.005). Four subjects without improvement with their abnormal baseline P-31 study and seven subjects with a normal baseline P-31 showed no significant differences in HEP response at follow-up (p = 0.3, p = 0.9, respectively). There was a modest degree of agreement between improvements in HEP response identified by P-31 MRS, and angina frequency from the SAQ with 78% of subjects with ischemia at baseline similarly classified (κ=0.571).

## Conclusions

This preliminary study demonstrates that Ranolazine treatment is associated with a significant reduction in myocardial ischemia in the majority of patients with angina at baseline.

